# Generation and maintenance of acentric stable double minutes from chromosome arms in inter-species hybrid cells

**DOI:** 10.1186/s12860-019-0186-3

**Published:** 2019-03-20

**Authors:** Noriaki Shimizu, Rita Kapoor, Shuhei Naniwa, Naoto Sakamaru, Taku Yamada, You-ki Yamamura, Koh-ichi Utani

**Affiliations:** 1 Graduate School of Biosphere Science, Hiroshima University, Higashi-hiroshima, Hiroshima, 739-8521 Japan; 2 Present address; Kanazawa Medical University, Uchinada, Japan

**Keywords:** Gene amplification, Double minutes, Cell fusion, Chromothripsis

## Abstract

**Background:**

Extrachromosomal acentric double minutes (DMs) contribute to human malignancy by carrying amplified oncogenes. Recent cancer genomics revealed that the pulverization of defined chromosome arms (chromothripsis) may generate DMs, however, nobody had actually generated DMs from chromosome arm in culture. Human chromosomes are lost in human-rodent hybrid cells.

**Results:**

We found that human acentric DMs with amplified c-*myc* were stable in human-rodent hybrid cells, although the degree of stability depended on the specific rodent cell type. Based on this finding, stable human-rodent hybrids were efficiently generated by tagging human DMs with a plasmid with drug-resistance gene. After cell fusion, human chromosomes were specifically pulverised and lost. Consistent with chromothripsis, pulverization of human chromosome arms was accompanied by the incorporation into micronuclei. Such micronucleus showed different replication timing from the main nucleus. Surprisingly, we found that the hybrid cells retained not only the original DMs, but also new DMs without plasmid-tag and c-*myc*, but with human *Alu*. These DMs were devoid of telomeres and centromeres, and were stable in culture for more than 3 months. Microarray analysis showed that the new DMs were generated from several human chromosomal regions containing genes advantageous for cellular growth. Such regions were completely different from the original DMs.

**Conclusions:**

The inter-species hybrid mimics the chromothripsis in culture. This is the first report that experimentally demonstrates the generation of multiple stable acentric DMs from the chromosome arm.

**Electronic supplementary material:**

The online version of this article (10.1186/s12860-019-0186-3) contains supplementary material, which is available to authorized users.

## Background

Gene amplification plays a pivotal role in human cell malignant transformation. Amplified genes locate at either chromosomal homogeneously staining region (HSR) or cytogenetically detectable large extrachromosomal elements called double minutes (DMs). The amplified genes in these structures confer a growth advantage or drug resistance, and are implicated in the development of various cancers (reviewed in [1–3]). Elimination of DMs from tumour cells leads to the reversion of malignant phenotypes and cellular differentiation, which underscored the importance of DMs for malignancy [4–6]. DMs and HSR are composed of the same amplicon, and inter-conversion between DMs and HSRs has been implicated in chemotherapy resistance of cancer cells [7].

Most DMs are telomere-negative, suggesting a circular structure. In addition, DMs are generally regarded as acentric, pending some exceptional case [8]. Despite their acentric nature, DMs stably segregate to daughter nuclei by sticking to normal chromosome arms during mitosis [9, 10]; this method of segregation, known as “hitchhiking”, is also utilised by various viral episomes [11–13], and it is the only known mechanism by which acentric elements are segregated to daughter nuclei after the cell division. DMs exhibit unique intracellular behaviour and are eliminated by specific mechanisms. Specifically, multiple DMs aggregate after DNA damage; following mitosis, the resultant aggregates generate cytoplasmic micronuclei that are subsequently eliminated from the cell (reviewed in [3, 14]).

Generation of DMs by premature chromosome condensation (PCC)-mediated chromosome arm pulverisation was first proposed three decades ago [15]. Recent whole-genome sequencing studies of several cancers suggested that pulverisation of defined chromosome arms (chromothripsis) generates complex rearrangements widely found in cancer chromosome arms. Such pulverization might contribute to the generation of DMs ([16] and reviewed in [17, 18]). It was shown that the pulverisation of specific chromosome arms was mediated by the incorporation of the chromosomes into micronuclei [19, 20]. On the other hand, previous studies have demonstrated that DMs can be generated by fragmentation of HSRs by cell fusion [21], by cutting HSRs with mega-endonuclease [22, 23] or by decreasing the DNA methylation level [24]. However, generation of complex DMs from normal chromosome arms, as predicted by chromothripsis, in cell culture have not been reported. Such an experimental system would support the chromothripsis, and mimic the event during the malignant transformation of human tumour.

We demonstrated previously that a plasmid harbouring a mammalian replication initiation region (IR) and a nuclear matrix attachment region (MAR) spontaneously initiates gene amplification and generates DMs and/or HSRs in transfected cells [25]. The experimental system appeared to mimic the ‘episome model’ of gene amplification [26]. Our study suggested that the plasmid is initially multimerised into a large circular extrachromosomal molecule in which the plasmid sequences are arranged as direct repeats [27–29]. If this multimerisation proceeds extensively, it might generate cytogenetically visible DMs [27, 28]. Alternatively, if the plasmid repeats are integrated into a chromosome arm, they can be further amplified to generate a HSR [28, 30]. The IR/MAR plasmid generates multiple DMs in human COLO 320 cells, but only rarely generates these structures when transfected into hamster CHO K1 cells [31]. This difference may reflect differences between these cell lines in the generation or stability of DMs. The initial aim of this study was uncovering whether the stability of DMs might be different between these cell lines or not.

Here, we fused human cells bearing DMs with rodent cells. Because human chromosome arms are specifically lost from human–rodent hybrid cells [32], we investigated whether the human DMs would be maintained in a rodent chromosomal background. The results suggested that the stability of DMs depends on the rodent cell type. We found that the human chromosome arms were lost through pulverisation, and serendipitously found that the pulverised chromatids generated new DMs de novo*,* as predicted by chromothripsis.

## Results

### The generation of extrachromosomal DMs from an IR/MAR plasmid is dependent on the host cell line

**Fig. 1:**
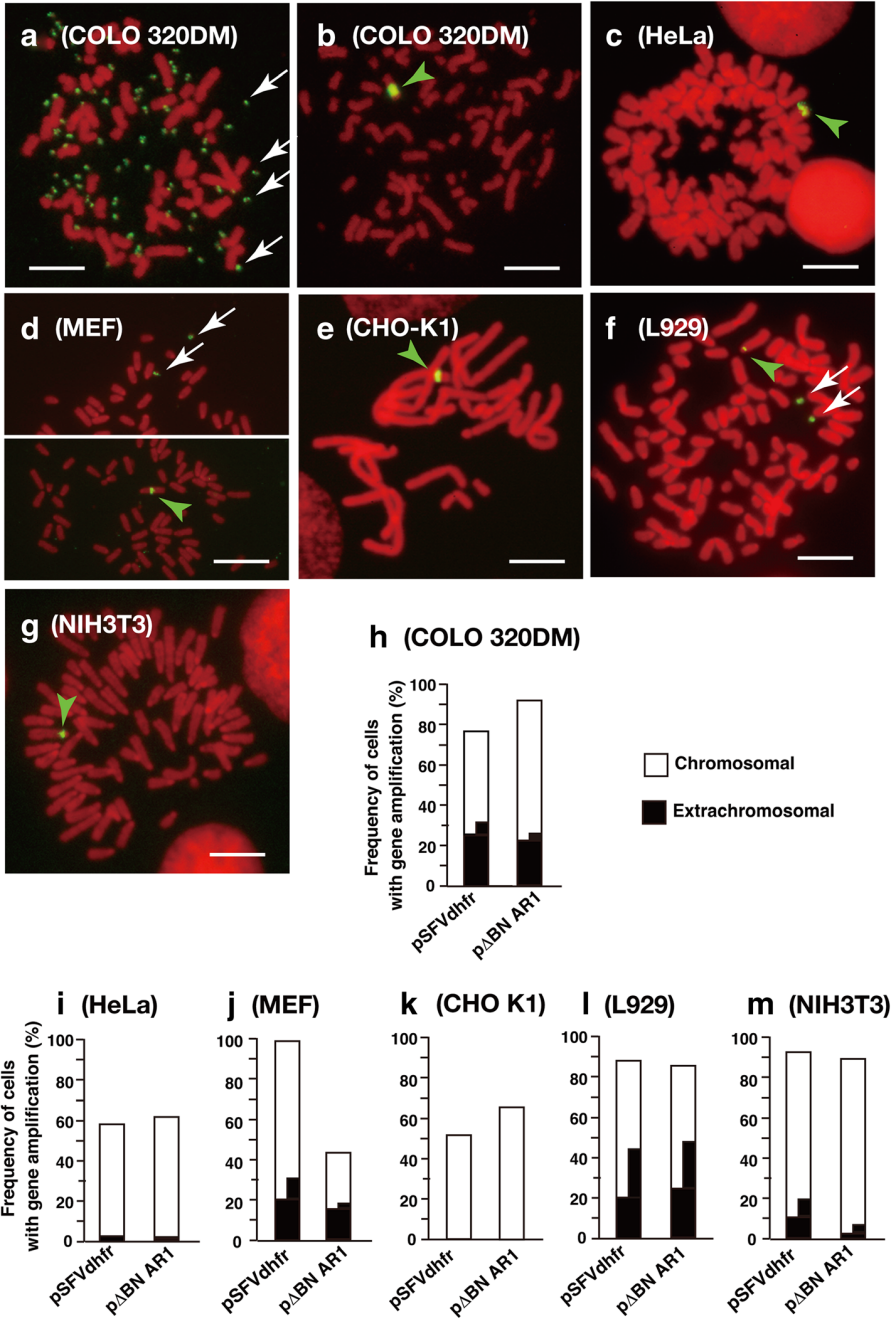
Generation of DMs from IR/MAR plasmids is dependent on the host cell line. **a**–**g** Representative images of IR/MAR plasmids (pSFVdhfr or p∆BN.AR1) after transfection into the indicated cell lines. After blasticidin selection of transfectants for 4–6 weeks, plasmid sequences were detected by FISH in metaphase spreads. The green arrowheads and white arrows indicate chromosomal and extrachromosomal amplification of the plasmid, respectively. Scale bar: 10 μm. **h**–**m** Frequencies of chromosomal (white) and extrachromosomal (black) amplification of plasmids in the transfected cell lines were determined by examining more than 30 metaphase chromosome spreads. Shown is a typical result. Quantitatively similar results were obtained from more than 30 (COLO 320DM), more than 5 (MEF, CHO K1), and more than 2 (HeLa, L929 and NIH3T3) independent transfections

Two different IR/MAR plasmids (pSFVdhfr and p∆BN.AR1) were transfected into two human (COLO 320DM and HeLa) and four rodent (MEF p53−/−, CHO-K1, L929, and NIH3T3) cell lines. After drug selection for approximately 1 month, the plasmid sequence was detected in metaphase spreads by fluorescence in situ hybridisation (FISH; Fig. 1). Consistent with our previous results, both of the IR/MAR plasmids were amplified at multiple extrachromosomal DMs and generated large chromosomal HSRs in COLO 320DM cells; however, they were rarely amplified at extrachromosomal sites in HeLa cells. In CHO K1 cells, weak plasmid signals were detected at chromosomal sites only, whereas the plasmids were amplified at both extrachromosomal and chromosomal sites in MEF, L929, and NIH3T3 cells; however, these cell lines contained fewer extrachromosomal DMs per cell than COLO 320DM cells. Thus, the presence of DMs was cell type-dependent and may reflect differential generation and/or maintenance of these structures.

### Establishment and characterisation of COLO 320 DM-donor cells

**Fig. 2:**
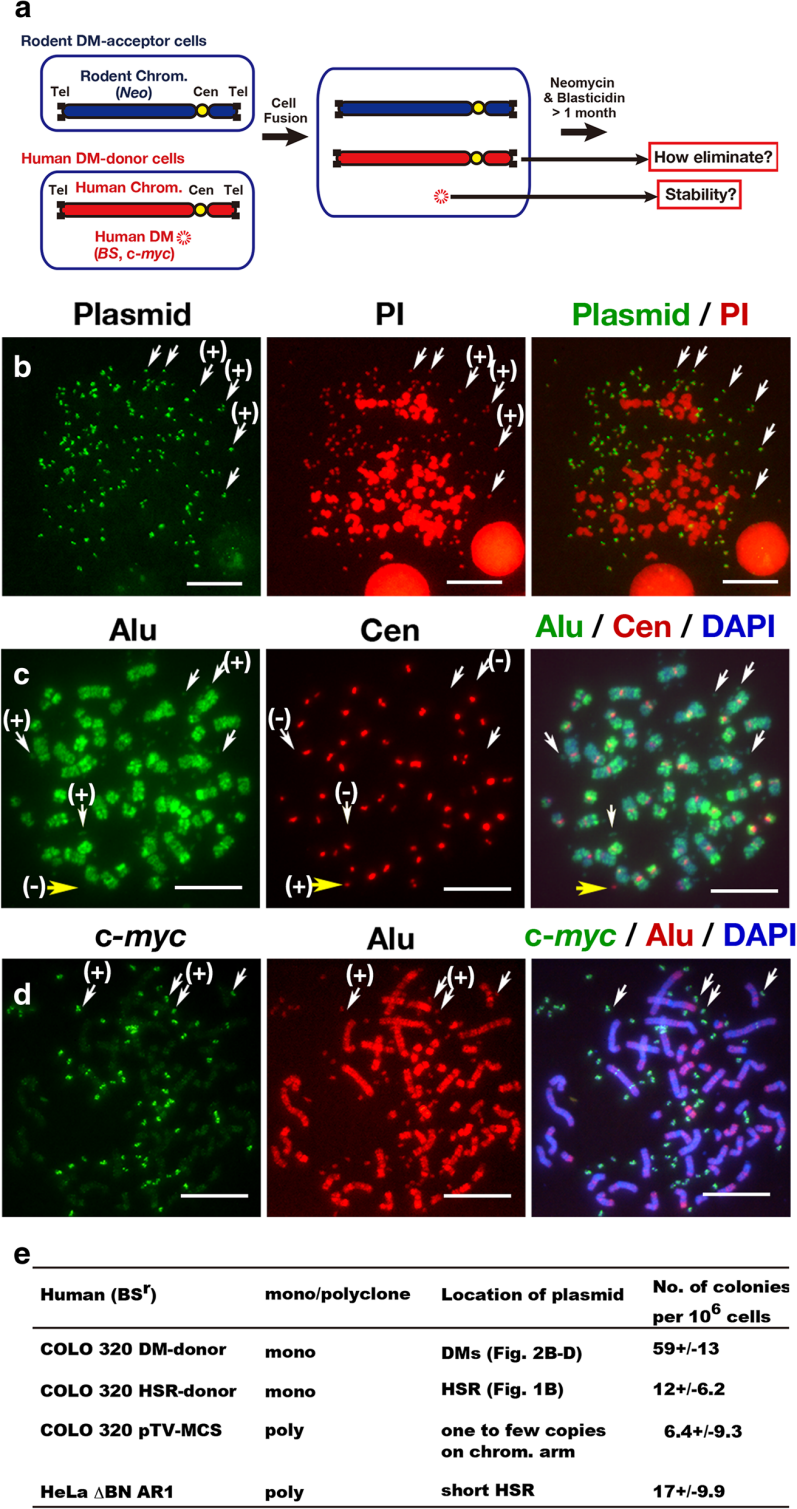
Experimental design and COLO 320DM-donor cells. **a** Graphical summary of the experimental design of this study. **b**–**d** Metaphase spreads from COLO 320DM-donor cells were hybridised with various probes. **b** Detection of IR/MAR plasmid sequences on all DMs in the COLO 320DM-donor cells. **c**, **d** Detection of human Alu (**c**, **d**) and c-*myc* sequences in the DMs in the COLO 320DM-donor cells (**d**). Most of the DMs were devoid of centromeric alphoid DNA (white arrows in c), although it was detected in a few DMs (yellow arrow in c). **c**-**d** Scale bar: 10 μm. **e** Frequencies of double-resistant colonies of monoclonal or polyclonal blasticidine-resistant human cell lines fused with neomycin-resistant mouse MEF acceptor cells. Colony number was calculated by examining three independent dishes containing 5 × 105 acceptor cells at the time of fusion. Mean +/− S.D. were calculated and are shown. Shown is a result from single experiment

Figure 2a schematically represents an experiment designed to clarify how human chromosome arms are lost after human–rodent cell fusion, and whether human DMs are also lost under such conditions. For this purpose, we established COLO 320DM-donor cells by tagging DMs in parental COLO 320DM cells via transfection with an IR/MAR plasmid harbouring a blasticidin resistance gene (*BS*). Because extrachromosomal molecules actively recombine with each other, the tandem repeats of the IR/MAR plasmid sequences recombined with all of the pre-existing DMs (Fig. 2b), consistent with our previous report [25, 27]. All the pre-existing DMs also contained human Alu sequences (Fig. 2c) as well as amplified c-*myc* genes (Fig. 2d). Hybridisation of the cells with a human pan-centromeric probe confirmed that most of the DMs were acentric (Fig. 2c); unexpectedly, however, a few DMs hybridised with the centromere probe. The average numbers of human centromere-positive DMs in the COLO 320DM-donor and parental COLO 320DM cell lines were 0.65 ± 0.75 and 0.3 ± 0.58 per cell, respectively (based on the analysis of at least 30 metaphase cells per group). These human centromere-positive DMs were apparently devoid of Alu sequences, suggesting that they were composed almost solely of the centromere sequence.

### Stable human–rodent hybrid cells are generated more frequently when a selection marker is located on human DMs

We first examined whether the presence of a drug-resistance gene on human DMs might influence the efficiency with which hybrid cells are obtained. To this end, we fused neomycin-resistant MEF acceptor cells with various human cells harbouring *BS* at different locations. The frequency of generation of double-resistant COLO 320DM-donor hybrid colonies, in which *BS* was located at the DMs, was approximately 10-fold higher than that of double-resistant COLO 320 pTV-MCS colonies, in which *BS* was located on the chromosome arm in low copy (Fig. 2e). Moderate numbers of double-resistant colonies were obtained when MEFs were fused with cells in which the *BS* -containing plasmid repeat was located within a chromosomal HSR (COLO 320 HSR-donor or HeLa ΔBN AR1). Under this condition, DMs might be generated by the fragmentation of HSR, as previously reported [21–24]. Overall, the results described above demonstrate that stable human–rodent hybrids could be obtained more efficiently if the selection marker was located at DMs in human cells.

### Human chromosome arms are specifically lost from human–rodent hybrid cells through pulverisation

**Fig. 3:**
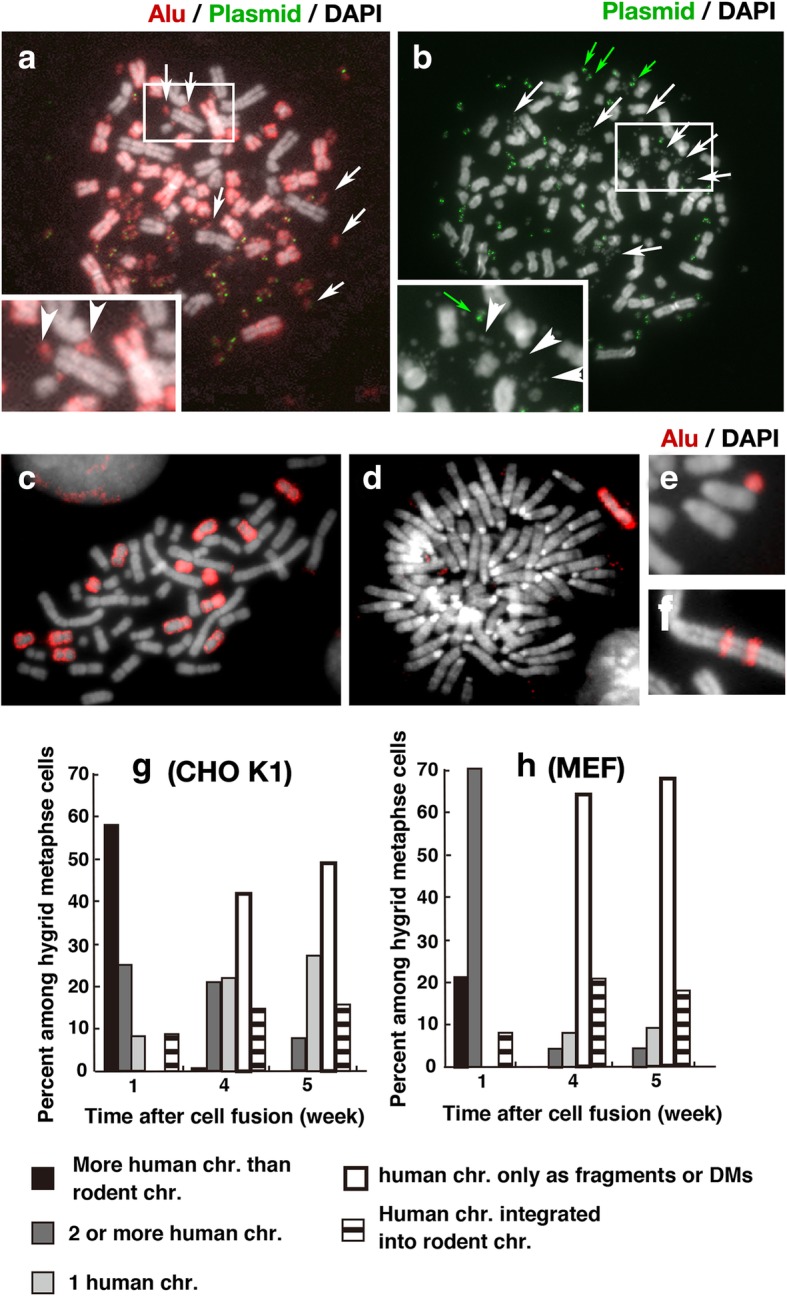
Human chromosomes are specifically lost from human–rodent hybrid cells through pulverisation. **a**–**f** Metaphase chromosome spreads of hybrids with CHO (**a**-**c**, **f**) or MEF (**d**, **e**) cells were prepared at 1 (**a**, **b**) or 4 weeks (**c**–**f**) after the fusion, and then hybridised with plasmid (**a**, **b**) and/or Alu (**a**, **c**-**f**) probe. Images show representative cells with more human chromosomes than mouse chromosomes (**a**), two or more human chromosomes (**c**), one human chromosome (**d**), human chromosome as fragments or DMs (**e**), and human chromosomes integrated into rodent chromosomes (**f**). The frequency of each case was scored by examining more than 30 metaphase cells at each time point and plotted in (**g**) and (**h**). Shown is a typical result. Qualitatively identical results were obtained from more than 3 independent fusion experiments. For panels A and B, the rectangular region was enlarged and shown as inset image. In these images, arrows indicate pulverised chromatids

After fusion with neo-resistant CHO K1 or MEF acceptor cells, human chromosome arms from the BS-resistant COLO 320DM-donor cells were rapidly lost. We prepared metaphase chromosome spread at 1, 4, and 5 weeks after fusion and double selection, and hybridised them with human Alu and plasmid probes. We could not analyse between 1 and 4 weeks, because number of viable cells was not sufficient for FISH analysis. One week after fusion, cells with more human chromosomes than mouse chromosomes predominated (Fig. 3a, g, h). By contrast, 4 or 5 weeks after the fusion, cells with fewer human chromosomes (Fig. 3c, d) or cells with only fragments or DMs derived from human chromosome arms (Fig. 3e) constituted the majority of the population (Fig. 3g, h). Some cells contained human chromatids integrated into rodent chromosome arms (Fig. 3f). Loss of human chromosome arms was faster in MEF hybrids than in CHO hybrid (Fig. 3g, h).

Importantly, 1 week after fusion, pulverised chromatids could be detected in DAPI-stained metaphase chromosome spreads (Fig. 3b), similar to the chromosome arm pulverisation observed during PCC. Hybridisation with an Alu probe revealed that these pulverised chromatids were mostly human-derived (Fig. 3a). A plasmid probe did not hybridise to most of the pulverised chromatids (Fig. 3a, b insets), suggesting that pulverisation of human chromosome arms had taken place.

### Human chromosome arms are specifically incorporated into the micronuclei and differentially replicated

**Fig. 4:**
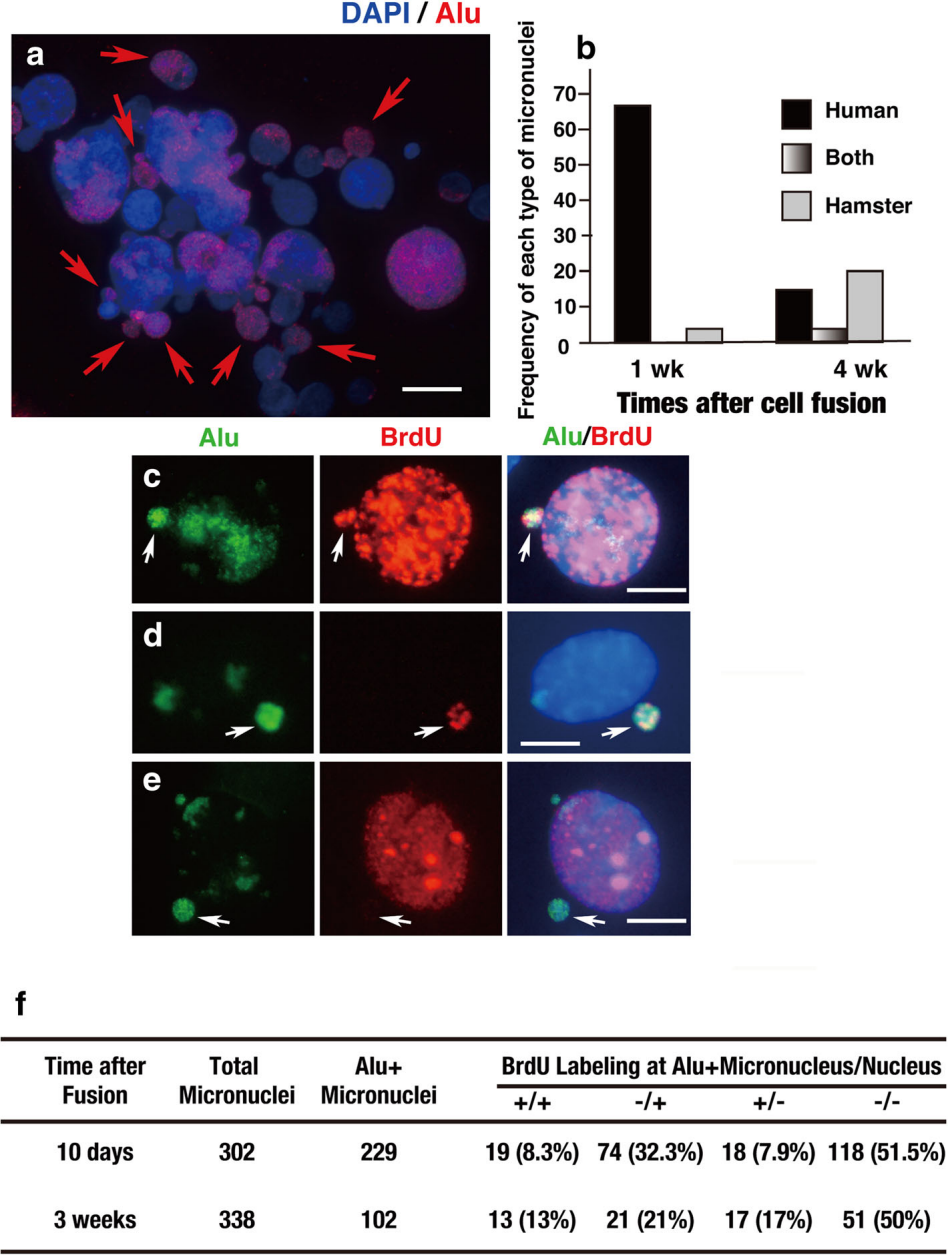
In human–rodent hybrid cells, human chromosomes are preferentially incorporated into micronuclei and replicated at a different time than the main nucleus. **a** FISH analysis performed 1 week after fusion of COLO 320DM-donor cells with CHO K1 acceptor cells, revealing that many Alu-positive human chromosomes were trapped in the micronuclei (arrows). Scale bar: 10 μm. **b** Frequencies of micronuclei containing human, hamster, or both types of chromosomes, based on scoring of more than 500 interphase nuclei at each time point. **c**–**e** BrdU 30 min pulse-labelling showing replication in both Alu-positive micronuclei and the main nucleus (**c**), the micronucleus only (**d**), or the cell nucleus only (**e**). Scale bars: 10 μm. **f** Frequencies of the replication events in the micronucleus and nucleus were calculated by examining the indicated number of total and Alu-positive micronuclei; data are summarised in the table. Shown is a typical result. Qualitatively identical results were obtained from 2 independent fusion experiments

When human chromosome arms were actively lost 1 week after fusion, approximately 70% of the hybrid cells contained micronuclei. Importantly, most of the micronuclei were composed of Alu-positive human chromatids (Fig. 4a, b). The frequency of such micronuclei had decreased significantly by 4 weeks after the fusion, when most of the human chromosome arms had already been lost.

By detecting pulse-incorporated BrdU among non-synchronous population, we compared the replication timing of Alu-positive micronuclei and the adjacent main nucleus at 10 days and 3 weeks after the cell fusion. The result revealed that a significant fraction of Alu-positive micronuclei replicated on a different time scale than the main nucleus (Fig. 4c–f). Such differential replication timing between the micronucleus and the nucleus might cause PCC of the micronuclear content, as reported [19].

### Maintenance of human DMs is dependent on the rodent acceptor cell line

**Fig. 5:**
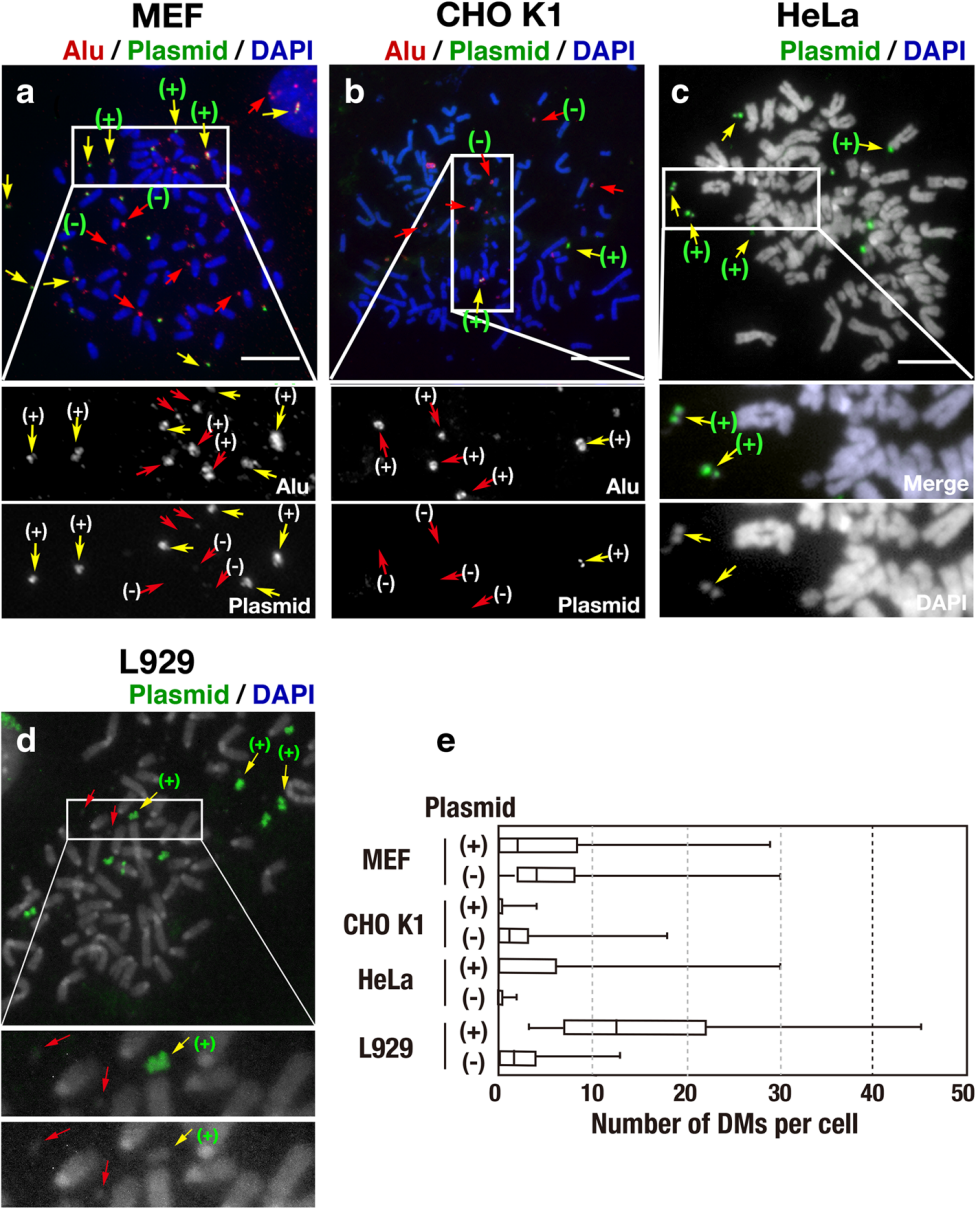
Various human DMs remain in the hybrid cells after pulverisation of human chromosomes. **a**-**d** Simultaneous detection of Alu and plasmid sequences (**a**, **b**) or detection of plasmid sequence (**c**, **d**) 4 weeks after fusion of COLO 320DM-donor cells with MEF (**a**), CHO-K1 (**b**), HeLa (**c**), or L929 (**d**) acceptor cells. DNA was counterstained by DAPI. DMs were identified by Alu-staining (**a**, **b**) or DAPI staining (**c**, **d**). Yellow and red arrows indicate DMs with and without the plasmid sequence, respectively. Scale bars: 10 μm. **e** Numbers of DMs with or without plasmid per hybrid cell. The box plot indicates the maximum and minimum (the right and the left end of the whiskers, respectively), first and third quartiles (both ends of the box), and median (line in the box). These values were obtained by examining 58 (MEF), 75 (CHO K1), 20 (HeLa), and 30 (L929) hybrid cells. Shown is a result from single experiment

One month after the fusion of rodent cells with COLO 320DM-donor cells, almost all the Alu-positive human chromosome arms had been lost from the hybrid cells. At that time, multiple Alu-positive human DMs were present among the rodent chromosome arms in stable hybrid cells (Fig. 5a, b and d). Unexpectedly, there were DMs with or without the plasmid sequence (Fig. 5a and b; yellow and red arrows, respectively), despite all DMs were originally tagged with the IR/MAR plasmid (Fig. 2b). By contrast, plasmid-negative DMs were barely detectable in human–human fusions of COLO 320DM-donor cells with HeLa acceptor cells (Fig. 5c and e), in which chromosome arm pulverisation was rare. It suggested that a portion of DMs might be generated de novo by pulverization of human chromosome arm, and it will be addressed in later section.

Interestingly, the number of DMs per cell was higher in MEF fusions or L929 fusions than in CHO fusions (Fig. 5e), suggesting that the DMs might be maintained more stably in the MEF or L929 background than in CHO cells. This finding is consistent with the previous finding that an IR/MAR plasmid generates DMs more frequently in MEF or L929 than in CHO cells (Fig. 1).

### Multiple DMs in the hybrids were centromere-negative and telomere-negative

**Fig. 6:**
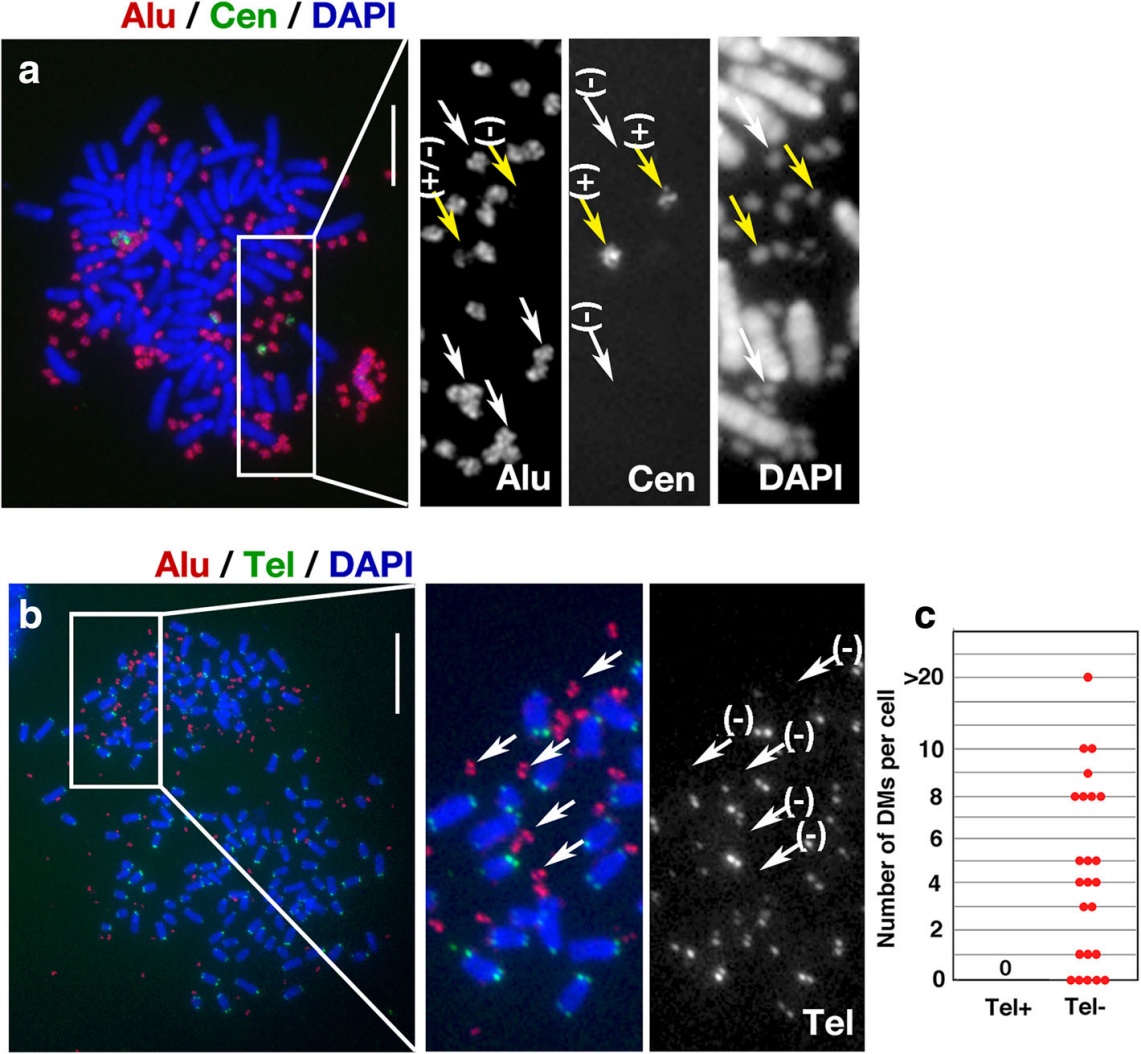
Human DMs with or without centromeres, but lacking telomeres, remained after human chromosome pulverisation. **a** 5 weeks after fusion of COLO 320DM-donor and MEF acceptor cells, metaphase chromosome spreads were prepared and simultaneously hybridised with a human pan-centromeric alpha satellite probe and an Alu probe. Representative image is shown. White and yellow arrows indicate DMs that are centromere-negative or -positive, respectively. **b** Simultaneous detection of Alu and telomere sequences in hybrid cells 5 weeks after fusion. The telomeric repeat sequence is conserved between human and mouse, and the FISH signal appeared at all chromosome ends. **c** Numbers of telomere-positive and -negative DMs per cell. Shown is a result from single experiment

We examined the presence of centromere sequences in the DMs in the COLO 320DM-donor × MEF-acceptor hybrid cells at 5 weeks after the fusion. Figure 6a shows a representative image. They contained multiple human DMs without centromeres (white arrows), as well as a few with centromeres (yellow arrows). Notably, the centromere-positive DMs rarely contained Alu-positive material (Fig. 6a, enlarged insets), consistent with the centromere-positive DMs in the original DM-donor cells (Fig. 2c). Therefore, they could easily be distinguished from many centromere-negative DMs based on the presence of Alu. Such centromere-positive DMs were interesting, however, we focused on the Alu-positive, centromere-negative DMs.

Next, we examined the presence of telomere sequences in the DMs from the COLO 320DM-donor × MEF-acceptor hybrid cells. Figures 6b shows a representative image. The telomere repeat sequence is conserved between human and mouse, and the telomere probe hybridised at both ends of all mouse chromosomes in metaphase spreads. Examination of 24 metaphase cells revealed the presence of multiple Alu-positive DMs; however, none of them exhibited a telomere signal (Fig. 6c). Consistent with this, DMs in human cancer are usually atelomeric [33, 34].

### Human DMs were generated de novo from several human chromosomal regions bearing growth-related genes

Presence of the plasmid-negative/Alu-positive DMs in the hybrid (Fig. 5) suggested that they might be generated de novo from human chromosome arms. Furthermore, in addition to the plasmid-negative DMs, there were Alu-positive/c-*myc*-negative DMs, nevertheless c-*myc* was amplified in original DMs (see below).

**Fig. 7:**
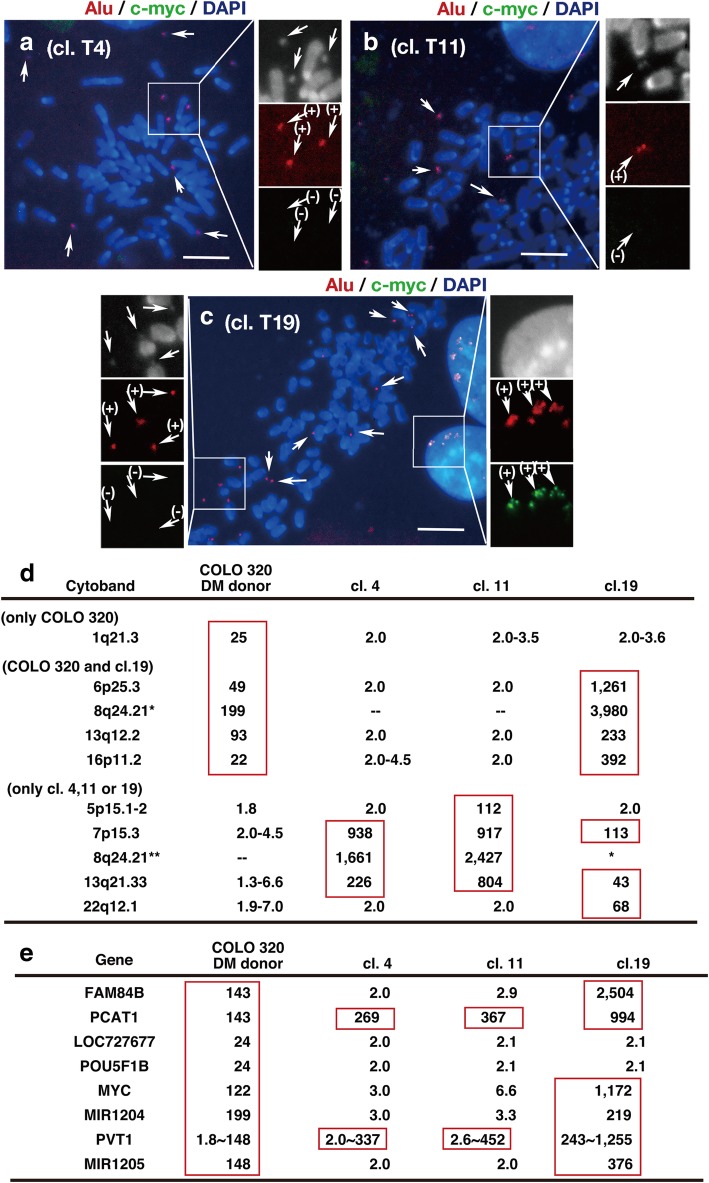
Microarray analysis of cloned hybrid cells reveals de novo generation of DMs from novel amplicons bearing tumorigenic genes. Hybrids of COLO 320DM-donor and MEF-acceptor cells were selected and grown under hypoxic (3% O_2_) conditions. From that culture, 24 clonal cell lines were obtained. Clones 4 (**a**) and 11 (**b**) had only c-*myc*-negative/Alu-positive DMs, whereas clone 19 (**c**) had both c-*myc*-positive and negative/Alu-positive DMs. The DNA from these cells, as well as DNA from COLO 320DM-donor and MEF-acceptor cells, were analysed by CytoScan™ HD Array; and the copy numbers per diploid cell for each cytoband were obtained. The copy number for the DNA from COLO 320DM-donor cells was standardised using data from normal human genomic DNA, and the copy number for the DNA from each clone was standardised using data from MEF-acceptor cells DNA. Panel **d** shows the cytobands that were amplified in at least one sample. Significantly amplified regions are boxed. 8q24.21 was amplified in all samples, but two different amplicons were detected (* and **). This is evident in panel **e**, where the copy numbers obtained for each gene within 8q24.21 are summarised. Shown is a result from single experiment

Therefore, we isolated cell clones with c-*myc*-negative/Alu-positive DMs. Namely, at 2 months after the fusion of COLO 320 DM-donor cells and MEF acceptor cells, we screened 24 randomly chosen clones by means of FISH to detect both c-*myc* and Alu sequence. Consequently, we identified three clones that contained human sequences almost exclusively in c-*myc*–negative/Alu-positive DMs (Fig. 7a to c). Clones T4 and T11 were composed solely of cells bearing such DMs (white arrows), whereas clone T19 was composed of a mixture of such cells and those bearing c-*myc*-positive/Alu-positive DMs, likely the original DMs (yellow arrows). The multiple DMs in these clones were homogeneous in size and shape. The DMs in these clones should be human centromere-negative, because all of the DMs were strongly Alu-positive. We further confirmed the DMs in clone T4 were actually centromere-negative (Additional file 1: Figure S1). Furthermore, the Alu-positive multiple DMs were stably maintained in these clones, because these clones were analysed at more than 2 months after the cell fusion, and such DMs were maintained during an additional 2 months of culture (Additional file 2: Figure S2).

We isolated total DNAs from these cells, as well from COLO 320DM donor and MEF acceptor cells, and subjected them to human microarray analysis to identify the amplified human sequences. Normal human genomic DNA and MEF acceptor cells were used as standards to evaluate amplification in COLO 320DM donor cells and each individual clone, respectively. Because of the differences between the mouse and human genomes, the amplification of human sequence in the mouse chromosomal background in these clones could have resulted in overestimation of the copy number; however, this did not prevent identification of the region of amplification. The raw data plots are shown in Additional file 3: Figure S3, and the cytobands that were amplified (boxed by a red line) in these samples are summarised in the table in Fig. 7d.

1q21.3 was amplified only in COLO 320DM donor cells, suggesting that the region was amplified at the chromosome arm in these cells. 6p25.3, 8q24.21, 13q12.2, and 16p11.2 were amplified in both COLO 320DM-donor cells and clone T19, and these cytobands should also be amplified in the original DMs in the COLO 320DM cells. This observation also suggested that the original DMs were maintained without alteration of the original amplicon in a portion of cells in clone T19. The amplification of these cytobands on DMs in COLO 320 cells was completely consistent with the published sequences of the DMs in COLO 320 cells [35]. On the other hand, clones 4, 11, and 19 had quite different amplicons. Specifically, 5p15.2 and 22q12.1 were amplified only in clones 4 and 19, respectively, and 7p15.3 and 13q21.33 were amplified in all three clones but not in COLO 320DM donor cells. 8q24.21 amplification was detected both in COLO 320DM-donor cells and in these clones; however, the amplicons were different, and we denoted them as 8q24.21* and 8q24.21**, respectively. Figure 7e shows the amplification of each gene within 8q24.21 in each sample. All genes were amplified in COLO 320DM-donor cells and in clone T19, whereas only a subset was amplified in clone T4 and T11; as expected from the FISH data, the copy number of c-*myc* (MYC) was lower in the latter two clones. Taken together, the results clearly showed that the DMs in these clones had amplicons that were different from those in the original DMs of COLO 320DM. Furthermore, the cytobands that were amplified from de novo generated DMs contained genes implicated in human malignancy. Specifically, 5p15.1–2 (amplified in clone T11) contained TRIO, 22q12.1 (amplified in clone T19) contained CHEK2, 7p15.3 (amplified in all clones) contained IL-6, and 13q21.33 (amplified in all clones) contained the upstream region of DACH1.

## Discussion

The specific loss of human chromosomes from human–rodent hybrid cells was discovered nearly 50 years ago [32]; however, the mechanism underlying this process has been poorly characterised in animal cells. Because of their utility in breeding, interspecies hybrids have been studied extensively in plants. In such cases, fertilisation of gametes from different species often results in uniparental chromosome loss. Mutation [36] or dissociation of centromeric histone H3 from the kinetochore [37] results in specific chromosome loss from the hybrid cell. Alternatively, chromosomes can be lost due to incomplete dissociation of cohesin during mitosis [38]. In any case, these abnormalities result in the generation of micronuclei containing the affected chromosome [38, 39]. A recent study reported chromosome arm shattering in micronuclei due to PCC [19] or replication anomalies [20] resulting from nuclear membrane rupture [40]. Here, we showed that human chromosomes in human–rodent hybrid cells were specifically incorporated into micronuclei and concomitantly pulverised. A most plausible explanation is that the human centromere might be less-active than the rodent centromere in such human-rodent hybrid cells. Whereas, acentric DMs were maintained in the hybrid.

Acentric DMs segregate to daughter nuclei by adhering to the mitotic chromosome arms (hitchhiking), and maintenance of human DMs among rodent chromosome arms suggests that hitchhiking may overcome the species barrier. Importantly, hitchhiking results in an unequal distribution of DMs between the daughter cells, potentially causing a dramatic increase in copy number under selective pressure. Notably, DMs appeared more frequently when human donor cells were fused to MEFs rather than CHO cells (Fig. 5), and the IR/MAR plasmid generated DMs more frequently in MEFs than in CHO cells (Fig. 1). This difference probably reflects cell type-specific differences in the efficiency of maintenance of acentric DMs by hitchhiking.

The stable hybrid cells contained not only the original c-*myc*-positive/Alu-positive and plasmid-tagged DMs, but also plasmid-negative/c-*myc*-negative/Alu-positive DMs. Microarray analysis suggested that the DMs in the former category had amplicons from 6p25.3, 8q24.21, 13q12.2, and 16p11.2. This result is consistent with the sequences of DMs in COLO 320 cells, in which these four regions are intermixed and amplified in a single circular structure [35]. Microarray analysis of the cells with the latter category of DMs revealed that these DMs were certainly generated de novo from the pulverised human chromosome arms, as demonstrated by the fact that the chromosome arm origins of these DMs were completely different from those of DMs in the former category. Moreover, these DMs were derived from a few chromosome arm regions, and most of them contained genes implicated in human carcinogenesis, e.g., TRIO, IL-6, PVT1, DACH1, and CHEK2 (see final section of Results). TRIO is involved in breast, ovarian, and prostate cancer, and its amplification is associated with urinary bladder cancer [41]. Overexpression of IL6 is tightly related to many tumours including breast, liver, lung, and prostate cancers, and its amplification has been reported in human glioblastomas [42]. In clones T4 and T11, a fragment of PVT1 was amplified, whereas MYC was not, indicating a difference relative to the amplicon in COLO 320DM. PVT1 encodes a long non-coding RNA; it is usually co-amplified with c-*myc* and is required for the expression of MYC and tumorigenesis of a wide variety of cancers [43]. CHEK2 and DACH1 is a tumour suppressor, however amplification of its mutated form may cause dominant-negative effect. Malfunction of CHEK2, a tumour suppressor, is involved in breast, ovarian, colorectal, osteocarcinoma, and prostate cancer. Suppression of DACH1 is also involved in breast and lung cancer. Because the multiple DMs in these stable clones were homogeneous in size and shape, it is likely that these regions were joined to a single structure and amplified on the same DMs in our clones; this is consistent with previous reports that DMs gather various chromosome arm regions together [35] and that recombination occurs frequently between extrachromosomal elements [3, 14]. Unequal segregation of acentric DMs during mitosis facilitates elevation of their cellular copy number. Thus, amplification of a certain gene on multiple DMs is possible when the gene confers a dose-dependent growth advantage.

Chromosome arm pulverisation inevitably generates fragments containing the IR and MAR sequences because vast numbers of these sequences are scattered throughout the genome. Circular DNA bearing the IR/MAR sequence undergoes gene amplification and generates multiple DMs (reviewed in [3]). Therefore, fusion of the IR/MAR with a gene that confers a growth advantage on cells leads to gene amplification and the generation of stable multiple DMs.

## Conclusions

Recent cancer genomics revealed that the pulverization of defined chromosome arms, chromothripsis, may generate DMs, however, nobody had actually generated DMs from chromosome arm in culture. The results presented here strongly suggested that the inter-species hybrid mimics the chromothripsis in culture. This is the first report that experimentally demonstrates the generation of multiple stable acentric DMs from the chromosome arm.

## Methods

### Plasmids

The origin and structure of pSFVdhfr were described previously [25]. This plasmid contains a blasticidin resistance gene (*BS*), a hygromycin resistance gene, and an IR (4.6 kbp) from the 3′-untranscribed region of the *DHFR* locus termed Ori β. The IR contains a sequence that exhibits in vitro MAR activity [25]. p∆BN.AR1 was constructed from pSFVdhfr by removing the hygromycin resistance gene and inserting a sequence from the mouse *Igκ* intron that exhibits strong MAR activity (AR1), as described previously [27]. The origins and structures of pSV2 ECFP-LacR (conventionally called pLacR-CFP) and pECMS2β were described in a previous study by our group [44]. The pLacR-CFP plasmid contains a neomycin resistance gene and a gene that expresses a fusion protein composed of a lactose repressor and enhanced cyan fluorescence protein. pECMS2β has a lactose operator array. pTV-MCS [45] has an AR1 MAR but no IR; hence, it was not amplified in transfected cells.

### Cell lines, culture, and transfection

COLO 320DM (human colorectal carcinoma) [25], HeLa (human cervical cancer) [25], and CHO-K1 (hamster ovary) [24] cells were cultured as described previously. COLO 320DM cells have multiple DMs containing amplified c-*myc* genes; the amplicon structure in these cells has been determined at the sequence level [35]. Mouse embryonic fibroblasts from *p53* gene knockout mice (MEF *p53*^−/−^) were cultured as described previously [46]; a population of MEF *p53*^−/−^ cells that immortalised spontaneously during long-term culture were used in this study. Mouse NIH3T3 (clone 5611, JCRB0615) and L929 (IFO50409) cells were obtained from the Japanese Collection of Research Bioresources and cultured in Dulbecco’s modified Eagle medium (DMEM) supplemented with 10% foetal calf serum. All transfections were performed using the GenePorter 2 Lipofection kit (Genlantis, San Diego, CA, USA). The stable transfectants shown in Fig. 1 were selected with 5 μg/ml blasticidin for approximately 1 month.

COLO 320DM-donor cells and COLO 320 HSR-donor cells were obtained by co-transfecting COLO 320DM cells with p∆BN.AR1 and pECMS2ß and selecting stable transfectants with 5 μg/ml blasticidin. We showed previously that co-transfection of cells with an IR/MAR-bearing plasmid (p∆BN.AR1 in this case) results in the co-amplification of any co-transfected sequence [27]. Using dual-colour FISH with probes specific to the *DHFR* IR and the lactose operator repeat, we obtained clonal cells in which the co-transfected sequences were co-amplified at multiple DMs (DM-donor cells) or at the chromosomal HSR (HSR-donor cells). The acceptor cell lines (HeLa LacR-CFP, MEF LacR-CFP, and CHO-K1-LacR CFP) were obtained by transfecting the parental cell lines with pLacR-CFP and selecting stable transfectants with 800 μg/ml G418. Clonal cell lines that exhibited moderate cyan fluorescence in the nucleus were identified and used in the study.

### Cell fusion and selection

In the experiments appearing in Figs. 2, 3, and 4, polyethylene glycol-mediated cell fusion was performed. Briefly, 2.5 × 10^6^ LacR-CFP acceptor cells and 2.5 × 10^6^ DM-donor cells were mixed, precipitated, washed once with serum-free acceptor cell medium, and pelleted by centrifugation. The cell pellet was loosened by tapping, and pre-warmed PEG1500 solution (1 ml of 50% in 70 mM HEPES, pH 8.0; Roche Diagnostics, GmbH, Germany) was added to the cells in a dropwise manner, followed by gentle mixing with a pipette tip. After 1 min, serum-free medium (15 ml) was added. The mixture was then centrifuged, and the cell pellet was suspended in serum-containing medium by gentle swirling. In the experiments appearing in Figs. 5, 6 and 7, cell fusion was mediated by Sendai virus HVJ-E protein using the GenomONE-CF kit (Ishihara Sangyo Kaisha, Ltd., Osaka, Japan). In this case, 1.4 × 10^5^ donor cells per well were centrifuged in a 6-well plate containing the same number of acceptor cells in each well. The fused cells were cultured and selected in acceptor cell medium containing 10% foetal calf serum, 10 μg/ml blasticidin, and 800–1000 μg/ml G418. Usually, colonies consisting of cells with the morphology of acceptor cells appeared after 2–3 weeks of selection. To obtain the clones mentioned in Fig. 7, we cultured and selected cells under an atmosphere of 3% O_2_ and 5% CO_2_ at 37 °C using a multi-gas incubator (MCO-5 M, Panasonic Healthcare Co.).

### FISH and cytochemical procedures

Preparation of chromosome spreads and the FISH procedure were performed as described previously [28]. The plasmid probe was prepared by labelling p∆BN.AR1 DNA with digoxigenin (DIG) or biotin using the BioPrime DNA Labelling Kit (Invitrogen, Waltham, MA, USA) with or without 10× DIG DNA Labelling Mixture (Roche, Basel, Switzerland). Residues 52–129 of the Alu consensus were amplified from human genomic DNA using PCR and the following primers: GCG GGC GGA TCA CTT GAG and GTA TTT TTA GTA GAG ACG GG. The PCR product had the same sequence as the synthetic Alu probe used for FISH [47]. The pan-centromeric probe was amplified from human genomic DNA using PCR and a primer set described previously [48]. The FITC-labelled protein nucleic acid probe used for telomere detection was purchased from FASMAC Co., Ltd. (Kanagawa, Japan).

*Microarray analysis.* Genomic DNA was isolated by the conventional method and hybridised using the human CytoScan™ HD Array Kit (Affymetrix Co.) and Reagent Kit (Affymetrix Co.). The data were analysed using the Partek® Genomics Suite® software (Partek Inc.); and the copy numbers per diploid cell for each cytoband were obtained. The copy number for the DNA from COLO 320DM-donor cells were standardised using data from normal human genome DNA. The copy number for the DNA from clone 4, 11, and 19 were standardised using the data from MEF acceptor cells. Because of differences between mouse and human sequences, the copy number for human genes in the mouse genomic background may have been overestimated. Nonetheless, the result should be qualitatively reliable with respect to the amplification of human sequences in these cells. The primary data were further analysed and plotted using Microsoft Excel®.

## Additional files

Additional file 1: **Figure S1.** The DMs in clone T4 were actually centromere-negative. Equal amount of clone T4 cells and COLO 320DM cells were mixed, simultaneously hybridized with Alu-probe and human centromere-probe and detected in red and green, respectively. DNA was counterstained by DAPI. (TIFF 1404 kb)

Additional file 2: **Figure S2.** The Alu-positive multiple DMs in clone T4 and T11 were stably maintained during 2 and 4 months after the cell fusion. Metaphase spreads were prepared from the indicated culture, and were analysed by FISH using Alu-probe. The number of Alu(+) DMs per cell was counted by examining 200 cells. The number increased during the culture, because they were acentric. (TIFF 1404 kb)

Additional file 3: **Figure S3.** Plots of raw data obtained from microarray analysis using human CytoScan™ HD Arrays. Data obtained from the analysis using the Partek® Genomics Suite® software was plotted in Excel. X-axis represents position along each chromosome, and each plot coincides the start position of the data. Y-axis represents copy number per cell; normal human genomic DNA and MEF acceptor cells were used as standards to evaluate amplification in COLO 320DM donor cells and each individual clone, respectively. (ZIP 3629 kb)

## Abbreviations

DM: Double minutes
HSR: Homogeneously staining region
IR: Replication initiation region
MAR: Matrix attachment region
