# Supplementary figures and images for: Generation and maintenance of acentric stable double minutes from chromosome arms in inter-species hybrid cells
Source: BMC Mol Cell Biol. 2019 Mar 20;20:2. doi: 10.1186/s12860-019-0186-3 (PMC6446505; doi:10.1186/s12860-019-0186-3)

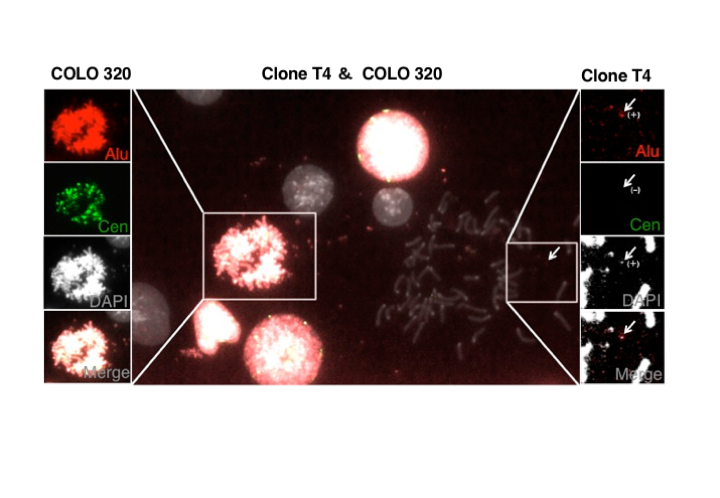

Supplement: Supplementary file 1 — Figure S1. The DMs in clone T4 were actually centromere-negative. Equal amount of clone T4 cells and COLO 320DM cells were mixed, simultaneously hybridized with Alu-probe and human centromere-probe and detected in red and green, respectively. DNA was counterstained by DAPI. (TIFF 1404 kb) [file 12860_2019_186_MOESM1_ESM.tiff]

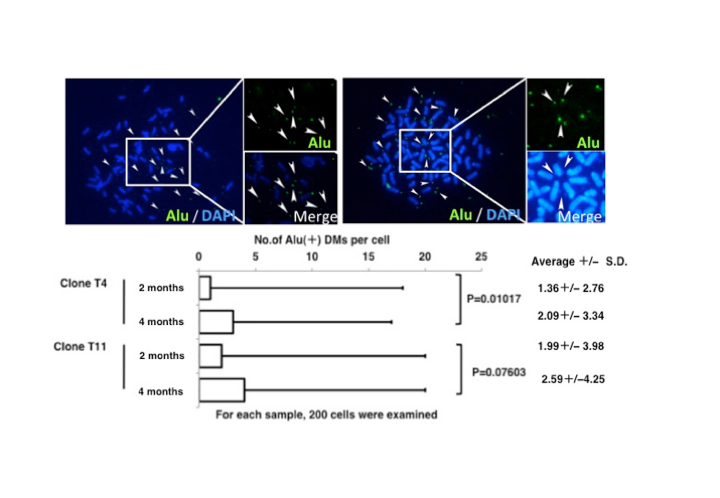

Supplement: Supplementary file 2 — Figure S2. The Alu-positive multiple DMs in clone T4 and T11 were stably maintained during 2 and 4 months after the cell fusion. Metaphase spreads were prepared from the indicated culture, and were analysed by FISH using Alu-probe. The number of Alu(+) DMs per cell was counted by examining 200 cells. The number increased during the culture, because they were acentric. (TIFF 1404 kb) [file 12860_2019_186_MOESM2_ESM.tiff]

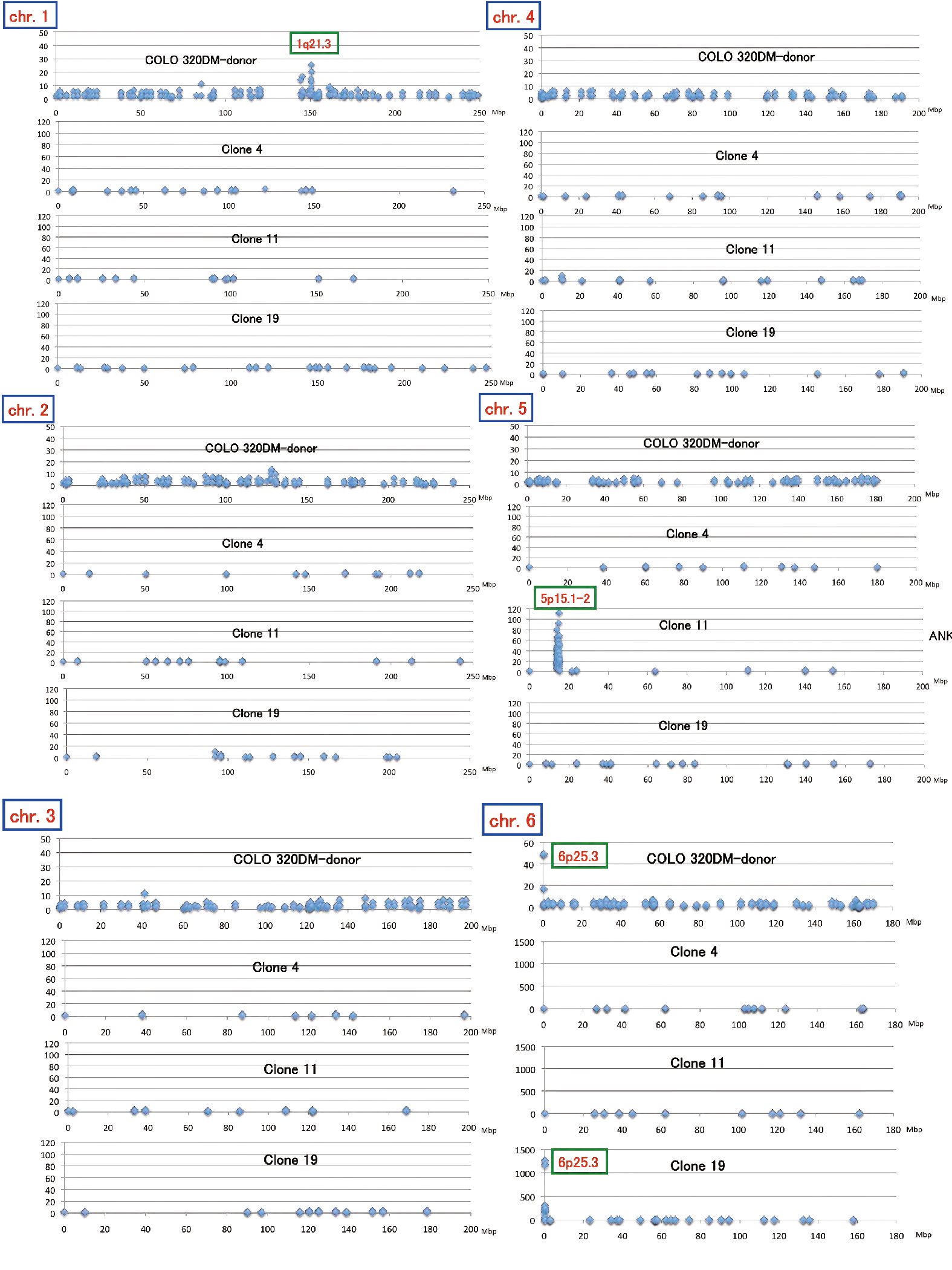

Supplement: Supplementary file 3 — Figure S3. Plots of raw data obtained from microarray analysis using human CytoScan™ HD Arrays. Data obtained from the analysis using the Partek® Genomics Suite® software was plotted in Excel. X-axis represents position along each chromosome, and each plot coincides the start position of the data. Y-axis represents copy number per cell; normal human genomic DNA and MEF acceptor cells were used as standards to evaluate amplification in COLO 320DM donor cells and each individual clone, respectively. (ZIP 3629 kb) [file 12860_2019_186_MOESM3_ESM.zip › Supp. Fig. S3_page_1.tiff]

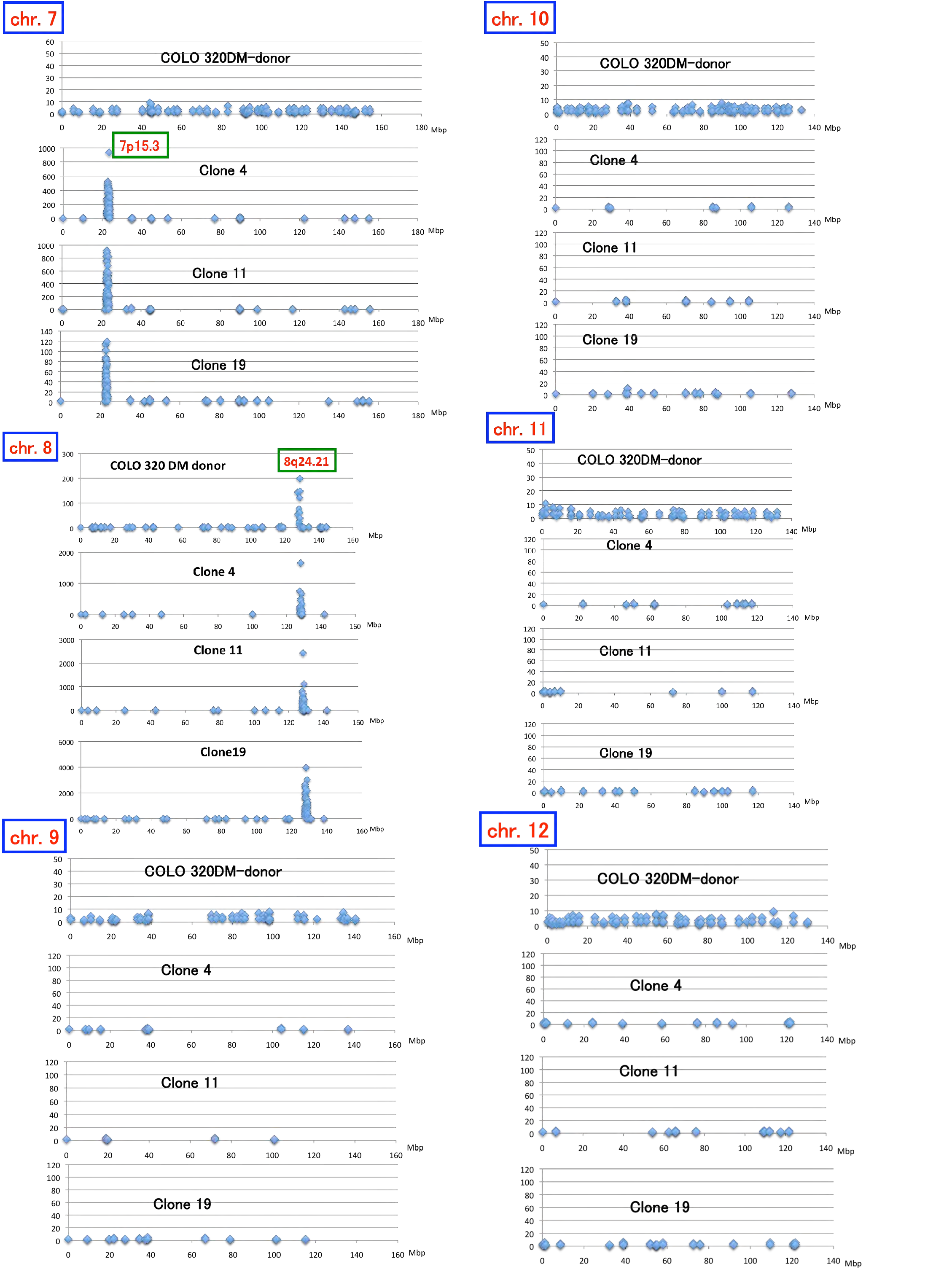

Supplement: Supplementary file 3 — Figure S3. Plots of raw data obtained from microarray analysis using human CytoScan™ HD Arrays. Data obtained from the analysis using the Partek® Genomics Suite® software was plotted in Excel. X-axis represents position along each chromosome, and each plot coincides the start position of the data. Y-axis represents copy number per cell; normal human genomic DNA and MEF acceptor cells were used as standards to evaluate amplification in COLO 320DM donor cells and each individual clone, respectively. (ZIP 3629 kb) [file 12860_2019_186_MOESM3_ESM.zip › Supp. Fig. S3_page_2.tiff]

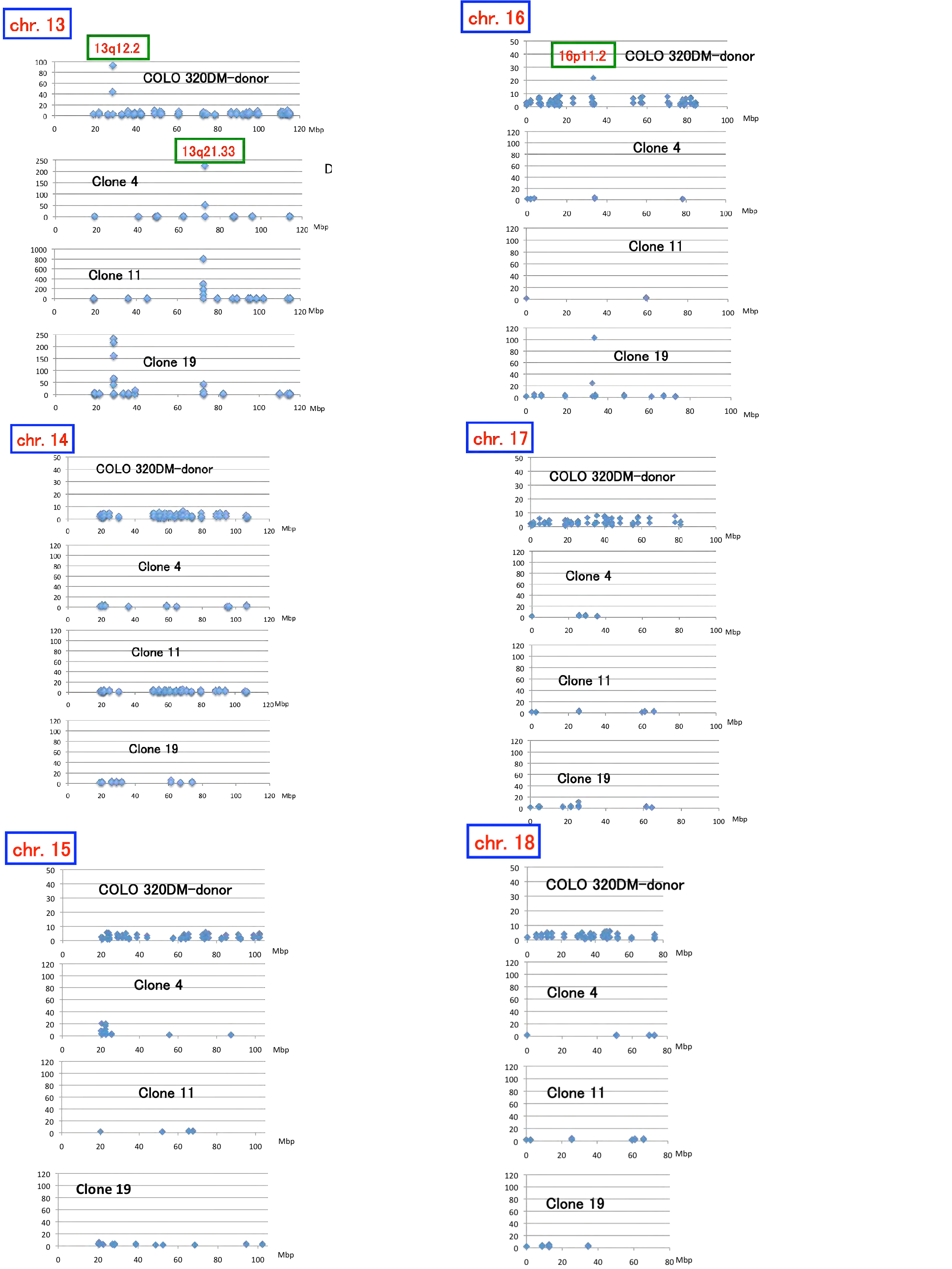

Supplement: Supplementary file 3 — Figure S3. Plots of raw data obtained from microarray analysis using human CytoScan™ HD Arrays. Data obtained from the analysis using the Partek® Genomics Suite® software was plotted in Excel. X-axis represents position along each chromosome, and each plot coincides the start position of the data. Y-axis represents copy number per cell; normal human genomic DNA and MEF acceptor cells were used as standards to evaluate amplification in COLO 320DM donor cells and each individual clone, respectively. (ZIP 3629 kb) [file 12860_2019_186_MOESM3_ESM.zip › Supp. Fig. S3_page_3.tiff]

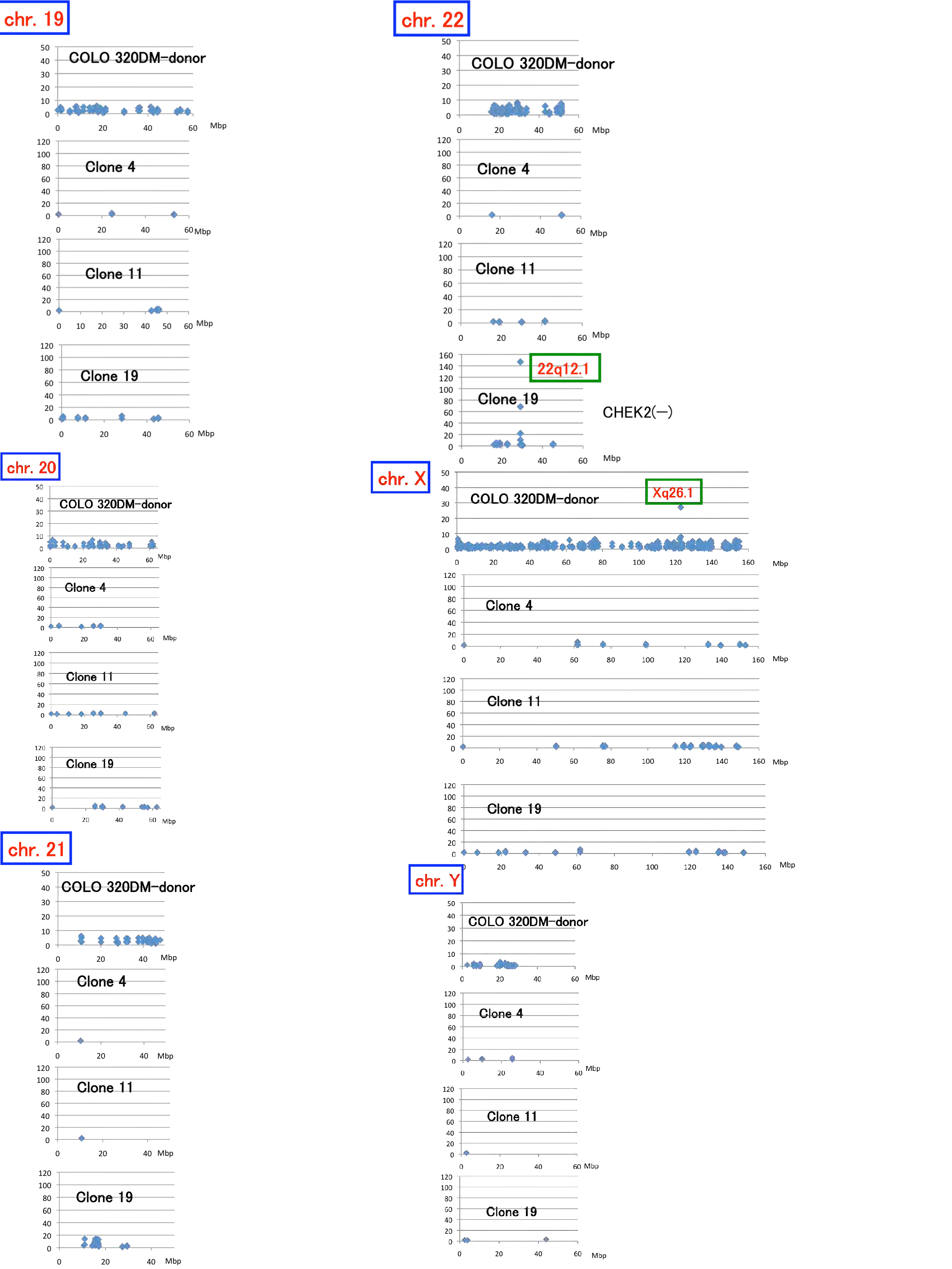

Supplement: Supplementary file 3 — Figure S3. Plots of raw data obtained from microarray analysis using human CytoScan™ HD Arrays. Data obtained from the analysis using the Partek® Genomics Suite® software was plotted in Excel. X-axis represents position along each chromosome, and each plot coincides the start position of the data. Y-axis represents copy number per cell; normal human genomic DNA and MEF acceptor cells were used as standards to evaluate amplification in COLO 320DM donor cells and each individual clone, respectively. (ZIP 3629 kb) [file 12860_2019_186_MOESM3_ESM.zip › Supp. Fig. S3_page_4.tiff]
